# Supplementary material for: Association between age at first alcohol use and heavy episodic drinking: An analysis of Thailand’s smoking and alcohol drinking behavior survey 2017
Source: PLoS One. 2021 Nov 8;16(11):e0259589. doi: 10.1371/journal.pone.0259589 (PMC8575245; doi:10.1371/journal.pone.0259589)
Supplement: Attachment — Submitted filename: Response to reviewer.docx [file pone.0259589.s001.docx]

**Response to Reviewers**

| **Reviewer #1** | |
| --- | --- |
| **Comment** | **Response** |
| **Major** |  |
| The analytic sample needs to be revised in order to accurately evaluate cross-sectional associations with age of first drink as defined. The use of the full sample (ages 15 and up) is completely accurate for estimating prevalence of HED and frequent HED among Thai-speakers in Thailand. However, I do not feel it is appropriate when evaluating associations between age at first drink and HED to include cases in the models that have not had the opportunity to be coded fully using the defined age of first drink measure: <20, 20-25, >25. Respondents aged 15-19 have to have an age of first drink at <20; those aged 20-25 do not have the ability to be coded as >25. This skews the analyses and resulting estimates. Since age of first drink was asked as an open-ended measure, I recommend that a series of models possibly be run. One possible approach would be that for respondents aged 15-19, include age at first drink as a continuous measure with controls that are appropriate for the age range (e.g., not including marital status). For respondents aged 20-25, possibly include age at first drink as a dichotomy of <20 vs. 20+. For respondents aged 26+, use the initial trichotomy of <20, 20-25, >25. This would also help address the “telescoping” that happens when attempting to remember age at first drink as one gets older. | - Three regression models by each age group were run and added to the manuscript as Table 4.  - The original Table 4 was re-numbered as Table 5. |
| An additional limitation that should be noted is that only Thai speakers were included; as the population in Thailand includes many hill tribes in various locations (particularly the North), findings may not generalize to these populations. | - This limitation was added. |
| In 2.3 HED, the wording of the item for HED reads, “How often have you drunk heavily (5 drinks)…”. Was this the actual wording used? Were females asked about the prevalence of 4 drinks? | - The item for HED in the survey followed the definition for males. Hence, male and female participants were asked with the same item for HED.  - We have corrected this point in 2.3.  - We have added the limitation concerning this point. |
| No mention of what was done to address missing data on covariates or outcome measures is available. Please state the level of missingness present (or at least a range), and what was done to address this, or if complete case data were used. | - Complete case data was used in the analysis. |
| I find it surprising that a yes/no item could be asked of exposure to alcohol advertising. Were respondents given any further information on what constitutes alcohol advertising? | - There is a single item about advertising exposure that is actually in a yes/no/unsure format. No further information was given to survey participants regarding alcohol advertising.  - The unsure response was coded as “no” in this analysis. We added this clarification. |
| Analyses should have included methods to address the complex sample (i.e., strata, clustering, weights). Please describe. | - All analysis was unweighted. |
| Please provide statistical testing of bivariate associations in Table 1. | - We thought this comment actually related to Table 2. P-value for bivariate association was added to Table 2. |
| **Minor** |  |
| The authors sometimes use “developed” and “developing” versus “higher-income” and “middle-income”. Please be consistent. | - To be consistent, “high-income” and “middle-income” were changed to “developed” and “developing”, respectively. |
| The authors should cite publications from national surveys for their prevalence estimates of nation-specific HID. Citation of a study looking only at HED among the Hispanic population at the US-Mexico border is not really appropriate for national HED prevalence, which can be found in sources such as the National Household Survey on Drug Use and the Monitoring the Future Survey. | - The citation was changed to the national survey as suggested. |
| The data presented on trends in current drinking among Thai adolescents is interesting, but confusing (p. 4). Please clarify. | - The sentences were revised for clarification. |
| In 2.2 Data source, the text reads 23,070 cases. However, in the sample flowchart, the total is 23,073. | - The correct number was 23,073. The text was corrected. |
| On p. 12, I believe the use of the word “attenuated” is incorrect, as this indicates the association was smaller for females than males. | - Table 5 (former Table 4) was modified and the paragraph was revised for clarification. |
|  | |
| **Reviewer #2** | |
| **Comment** | **Response** |
| The background introduction may be further strengthened by informing readers a little about the social/cultural background in Thailand and how that may contribute to the discrepancy in heavy drinking prevalence between Thailand and developed countries. | A paragraph was added to the introduction about prevalence of drinkers, cultural background related to drinking, and alcohol control measure. |
| Methods section: A little more specifics in the introduction of the database and sample. In particular, what is the proportion of the missing? | - We added the information on the response rate of the survey.  - A number of total samples in this analysis is 23,073. Complete case data was used in the multivariate analysis. The number of sample included in each specific analysis was in the header row or title of each table. The difference between 23,073 and the number indicated in the header row was incomplete cases that were excluded. |
| Methods section: Is the age category of >=25 relative to the other two younger groups too broad? | An average age at first alcohol use was 20.6. Less than 20% of participants started drinking ≥25 years old. Hence, we did not modify this variable. |
| Result: It is better to provide specific statistics when presenting the results in the texts to facilitate reading | Statistics were added as suggested. |
| Result: Given that this is a cross-sectional study, it is better to say correlates rather than “Determinants of HED and frequent HED” | The word “determinants” was changed to “factors associated with …” |
| Result: The statement regarding the interaction between gender and initial alcohol use doesn’t seem to match the findings in Table 4 and is in contrast with its following statement: “however, female sex attenuated the effect of age at first alcohol use on HED and frequent HED. For example, in females, the effect of age at first alcohol use <20 years on HED and frequent HED was increased by 66% and 103%, respectively.” Female earlier onset seems to increase the risk of HED instead. | - Table 5 (former Table 4) was modified and the paragraph was revised for clarification. |
| Discussion: The discussion can be further enriched by more closely relating the findings to relevant literature. For example, evidence concerning gateway theory in substance use in general may help strengthen the discussion from early drinking onset to HED (e.g., Zhang, S., Wu, S., Wu, Q., Durkin, D. W., & Marsiglia, F. F. (2021). Adolescent drug use initiation and transition into other drugs: A retrospective longitudinal examination across race/ethnicity. Addictive Behaviors, 113, 106679.) | This point was added to the discussion. |
|  | |
| **Reviewer #3** | |
| **Comment** | **Response** |
| **Major** |  |
| Review the use of the term gender, as apparently the variable used was sex. For this, please consider the SAGER guidelines (https://www.equator-network.org/reporting-guidelines/sager-guidelines/) | - The word “gender” is replaced by “sex”. |
| Emphasize, in the discussion, the main differences in the results observed in Thailand in relation to other countries, in order to reinforce the importance of carrying out the study. Also inform about public policies to combat the harmful use of alcohol in force in Thailand, and how the results of the study can contribute to the improvement of such policies. | - We added the discussion about the effect of drinking location, which somewhat differed to previous findings. |
| **Minor** |  |
| Title: The title should include the name of the country where the survey was conducted. | The title was modified as suggested. |
| Abstract: - Please, use the same objective, as stated at the end of the introduction: “This study aimed to investigate the association between age at first alcohol use and HED using data from a nationally representative survey in Thailand.” | The objective in abstract was changed to be identical to the sentence in the introduction. |
| Abstract: - The abstract should show effect sizes and confidence intervals. “Regular drinking, drinking at home, and exposure to alcohol advertising increased the likelihood of HED.” Also, it is suggested to replace “likelihood” by “chance”, which is more adequate to the type of analysis used (logistic regression). | - The abstract was revised as suggested. |
| Abstract: - “There was a significant interaction between the effect of age at first alcohol use and gender on HED and frequent HED.” Please, indicate the direction of the associations, e.g., lower or higher age? Male or female? | - The abstract was revised as suggested. |
| Abstract: “This study provides evidence from middle-income countries that early onset of alcohol use is associated with HED.” – a middle income country | - The sentence was revised as suggested. |
| Keywords: - Please include a keyword related to study design (cross-sectional studies) | - “Cross-sectional study” was added as a key word |
| Introduction: - It should include data on HED prevalence in Thailand. Data on the burden of disease caused by alcohol could also be included. | - HED prevalence in Thailand was added. |
| Methods: - At the “study design” subsection, please indicate clearly the study design (cross-sectional) | - It was revised as suggested. |
| Methods: - Please, review the use of the word “gender”. | - The word “gender” is replaced by “sex”. |
| Methods: - Please, explain how “exposure to alcohol advertising” was measured and what was considered as “alcohol advertising”. | Explanation was added for clarification. |
| Methods: - Statistical analyses should include the criteria used for including and maintaining variables in the adjusted regression model. | - Explanation was added for clarification |
| Results: - The higher prevalence of HED while drinking at home could be emphasised in the text. | - We added the discussion about the effect of drinking location, which somewhat differed to previous findings. |
| Results: - Tables 3 and 4: please, indicate as footnote the name of the statistical tests used to calculate the p-values. | - This was added as suggested. |
| Discussion: At the beginning of this section, sex differences could also be mentioned. Also, sex differences could be more discussed. The discussion could be deepened and include dialogue with studies carried out with other middle-income countries, including Asian countries, and countries in other continents, such as Brazil, Mexico, South Africa. | - We added the discussion as suggested. |
