# Supplementary material for: Association between age at first alcohol use and heavy episodic drinking: An analysis of Thailand’s smoking and alcohol drinking behavior survey 2017
Source: PLoS One. 2021 Nov 8;16(11):e0259589. doi: 10.1371/journal.pone.0259589 (PMC8575245; doi:10.1371/journal.pone.0259589)
Supplement: Attachment — Submitted filename: Response to reviewer.docx [file pone.0259589.s002.docx]

**Response to Editor Comments:**

| **Comment** | **Response** |
| --- | --- |
| - English edit of the manuscript | The manuscript was re-edited by a professional editing service. The certificate is uploaded with this submission. |
| - the p values on the tables are sometimes not well placed. A p value for each category of answer is expected when when you have a variable with 3 or more categories (for example: age: 15-19, 20-24 and 25+ - here you should have 2 p values: 15-19 is the reference and 20-24 has one p value and 25+ has another p value). Most of the tables presents at least one error on the report of the p values). | - Table 2: p-values were computed from chi-squared test, which tests an overall effect of a factor; hence, it has only one p-value per one factor.  - Table3, 4, 5: for each factor, logistic regression employed two statistical tests—Wald’s test and LR test. LR test tests an overall effect of a factor, while Wald’s test tests an effect of each factor’s level. Originally, only p-values from LR test were reported. According to editor’s suggestions, p-values from Wald’s test were added to Table 3-5. The table heading was modified to reflect this change. |
| - include in the methods section that missing data was excluded and that the analysis refers to complete cases only. | The sentence was added to 2.2 of the methods section. |
